# Attractiveness of Host Plant Volatile Extracts to the Asian Citrus Psyllid, *Diaphorina citri,* is Reduced by Terpenoids from the Non-Host Cashew

**DOI:** 10.1007/s10886-018-0937-1

**Published:** 2018-03-02

**Authors:** Marilene Fancelli, Miguel Borges, Raul A. Laumann, John A. Pickett, Michael A. Birkett, Maria C. Blassioli-Moraes

**Affiliations:** 1 Embrapa Cassava and Fruits, PO Box 007, Cruz das Almas, 44380-000 Brazil; 2 Embrapa Genetic Resources and Biotechnology, Brasília, 70770-917 Brazil; 3 Rothamsted Research, Harpenden, Hertfordshire, AL5 2JQ UK

**Keywords:** Asian citrus psyllid, Plant/insect interactions, Host, Non-host, Cashew, Terpenoid, DMNT, TMTT

## Abstract

*Diaphorina citri* is a vector of the bacterial causative agent of Huanglongbing (HLB = Citrus greening), a severe disease affecting citrus crops. As there is no known control for HLB, manipulating insect behaviour through deployment of semiochemicals offers a promising opportunity for protecting citrus crops. The behavioural responses of *D. citri* to plant volatiles, and the identity of these plant volatiles were investigated. Volatiles were collected from host plants *Murraya paniculata*, *Citrus sinensis*, *C. reshni*, *C. limettioides, Poncirus trifoliata,* and from non-host plants *Psidium guajava*, *Mangifera indica*, *Anacardium occidentale*. In behavioural assays, female *D. citri* spent more time in the arms containing volatiles from either *M. paniculata* or *C. sinensis* compared to the control arms. When *D. citri* was exposed to volatiles collected from *A. occidentale*, they preferred the control arm. Volatiles emitted from the other studied plants did not influence the foraging behaviour of *D. citri*. Chemical analyses of volatile extracts from *C. sinensis, M. paniculata,* and *A. occidentale* revealed the presence of the terpenoids (*E*)-4,8-dimethylnona-1,3,7–triene (DMNT) and (*E*,*E*)-4,8,12-trimethyltrideca-1,3,7,11-tetraene (TMTT) in higher amounts in *A. occidentale*. In further behavioural bioassays, female *D. citri* spent less time in arms containing a synthetic blend of DMNT and TMTT compared to the control arms. Female *D. citri* also spent less time in arms containing the synthetic blend in combination with volatile extracts from either *M. paniculata* or *C. sinensis* compared to the control arms. Results suggest that higher release of the two terpenoids by *A. occidentale* make this species unattractive to *D. citri*, and that the terpenoids could be used in reducing colonisation of citrus plants and therefore HLB infection.

## Introduction

The Asian citrus psyllid, *Diaphorina citri* Kuwayama (Hemiptera: Liviidae), is a vector of the bacterial causative agent of Huanglongbing (HLB = Citrus greening), a disease that is currently the greatest global threat to citrus production (Bové 2006; da Graça 1991; Halbert and Manjunath 2004). As there is no effective control for this disease (Bové 2006; Halbert and Manjunath 2004), citrus growers rely on the monitoring and eradication of symptomatic trees, the use of healthy citrus plants from certified nurseries, and management of the vector (Belasque et al. 2010; Gottwald et al. 2012). Suppression of *D. citri* populations is mostly based on chemical control (Boina et al. 2010; Sétamou et al. 2010; Tiwari et al. 2012). However, alternative control measures have been investigated, including essential oils, botanical insecticides, plant-derived semiochemicals (Borad et al. 2001; Mann et al. 2011; Patt and Sétamou 2010) and biological control (Hoddle 2012; Juan-Blasco et al. 2012; Stauderman et al. 2012). Essential oils and plant-derived semiochemicals have been deployed to manipulate the behaviour of natural enemies for pest management, being released from either synthetic lures or from companion plants as described in push-pull systems for managing stemborer pests (Blassioli-Moraes et al. 2013; Khan et al. 2014; Pickett et al. 2014).

Several previous studies have investigated the chemical ecology of interactions between *D. citri* and its host plants. Numerous members of the Rutaceae are considered attractive to *D. citri*, e.g. orange jasmine, *Murraya paniculata* L., curry leaf tree, *Bergera koenigii* L. and alemow, *Citrus macrophylla* Wester (Patt and Sétamou 2010; Wenninger et al. 2008, 2009; Westbrook et al. 2011). Differences in host plant suitability for the vector, and disease susceptibility, have been reported, e.g. Cleopatra mandarin, *C. reshni* Hort. and Japanese bitter orange, *Poncirus trifoliata* L. Rafinesque have been cited as unsuitable hosts for *D. citri* development, survival, and reproduction (Tsagkarakis and Rogers 2010; Westbrook et al. 2011), whilst *P. trifoliata* and Palestinian sweet lime, *C. limettioides* Tanaka, have been cited as extremely tolerant and tolerant to HLB, respectively, under greenhouse conditions (Folimonova et al. 2009). *M. paniculata* is also considered a transitory host for causative agents of HLB (Manjunath et al. 2008). The volatile profiles of host Rutaceae plants differing in susceptibility to *D. citri* have been investigated, and qualitative differences between profiles have been observed (Robbins et al. 2012). However, the influence of volatiles on foraging behaviour of *D. citri* has not been evaluated (Robbins et al. 2012).

The location and selection of suitable host plants by insect pests is mediated by olfactory perception of blends of volatile semiochemicals (Bruce et al. 2005). For *D. citri*, preference of specific blends that indicate host suitability can potentially elicit differential host searching between suitable and unsuitable host plants. To test the hypothesis that host volatile blends impact *D. citri* host seeking behaviour, interactions between *D. citri* and volatiles collected from a range of suitable, unsuitable, and non-host plants were studied. *M. paniculata* and sweet orange, *C. sinensis* (L.) Osbeck were selected as most attractive host plants (Ikeda and Ashihara 2008; Patt and Sétamou 2010; Borgoni et al. 2014), with *C. reshni*, *C. limettioides,* and *P. trifoliata* being selected as less attractive hosts (Tsagkarakis and Rogers 2010; Westbrook et al. 2011). Previous work reported that guava, *Psidium guajava* L. (Myrtaceae), leaves and volatiles repel *D. citri* and hamper location of Rangpur (lemandarin), *C. limonia* Osbeck (Rouseff et al. 2008; da Silva et al. 2016; Zaka et al. 2010). Therefore, guava was selected for study here as a potential non-host. Mango, *Mangifera indica* L. (Anacardiaceae) and cashew, *Anacardium occidentale* L. (Anacardiaceae) were also selected for investigation as non-hosts because they are economically important crops and easily cultivated in the same areas where citrus plants are grown. The work here provides the underpinning science for the development of a push-pull strategy for the management of *D. citri,* and therefore HLB, in Brazilian citrus crops.

## Methods and Materials

### Insect and Plant Material

*D. citri* rearing was started from a healthy population collected in the province of Tien Giang, Vietnam and kept in quarantined conditions by CIRAD, Montpellier, France since 2005. The insects were reared on *Murraya exotica* (L.) Jack in a quarantined insectary (27 °C; 70% RH; 14:6 h L:D regime) at Rothamsted Research, Harpenden, Hertfordshire, UK. Sleeve glass cages (60 cm height × 40 cm wide × 40 cm depth) were used for confining the insects on the plants. *M. exotica* plants were grown in a glasshouse (25 °C; 16:8 h L:D regime). Cuttings obtained from *M. exotica* developed plants were dipped in a powder hormone and placed into a perlite filled pot until they root, which lasted around 6 to 9 weeks. Afterwards, they were transplanted into a compost enriched soil filled pot until they started producing young shoots, when they were transferred to the cages for insect rearing. The plants used for volatile collection were obtained from Embrapa Cassava and Fruits, Cruz das Almas, BA, Brazil and kept under screened greenhouse to prevent damage by arthropods.

### Volatile Collection

Dynamic headspace collection was used to sample volatiles from host plants i.e. orange jasmine, *Murraya paniculata*, sweet orange Pera D6, *Citrus sinensis*, Cleopatra mandarin, *C. reshni*, Palestine sweet lime, *C. limettioides,* and Japanese bitter orange, *Poncirus trifoliata*, and non-host plants i.e. guava, *Psidium guajava*, mango, *Mangifera indica,* and cashew, *Anacardium occidentale*. Considering differences in the size of plants, efforts were made to utilise them when they were between 20 and 40 cm in height. This size was chosen because the plants possessed four or more complete leaves. Plants were placed individually inside cylindrical glass chambers (internal volume 10 l) as described by Michereff et al. (2011). The pots and soil were wrapped with aluminium foil to minimize interference of volatiles from these sources. A system of 12 independent chambers was run simultaneously with plant species (*N* = 5 individuals of each species) randomly distributed in a total of four runs in consecutive days. Volatiles were collected for 24 h. A single glass tube containing 60 mg of the adsorbent Super Q (80–100 mesh, Alltech, PA, USA) was inserted into each of the chambers and was also connected via PTFE tubing to a vacuum pump with the airflow set to 0.6 l/min. Simultaneously, charcoal-filtered air was pushed into the chamber at an airflow of 1.0 l/min, creating an overall positive pressure in the chamber to minimise contamination. The trapped volatiles collected in a 24 h period were eluted from the adsorbent using 500 μl of *n*-hexane and the collected extracts pre-concentrated to 100 μl under a gentle flow of N_2_. The samples containing the volatiles were kept at −20 °C until use in bioassays and chemical analysis. Super Q tubes were conditioned before use by washing with redistilled dichloromethane (1 ml) and *n*-hexane (1 ml) and heating in an oven (132 °C) under a stream of nitrogen for 2 h.

### Four-Arm Olfactometer Bioassay

A Perspex olfactometer (12 cm diameter) (Webster et al. 2010) was used to determine the behavioural responses of *D. citri* females to the volatiles collected from *Murraya paniculata*, *Citrus sinensis*, *C. reshni*, *C. limettioides,* and *Poncirus trifoliata* and non-host plants *Psidium guajava*, *Mangifera indica,* and *Anacardium occidentale*. Bioassays were carried out under controlled conditions (24 ± 2 °C, 60% RH) using a four-arm olfactometer (Pettersson 1970) provided with an exhaustion system. One treated arm (volatile extract containing the plant volatiles or a synthetic blend solution) was compared against three control arms (*n*-hexane). Charcoal-filtered air was drawn from the olfactometer at the rate of 200 ml min^−1^. For each experiment, a single 4–7 day old adult female, starved for 1 h, was released into the central area of the olfactometer through a small hole (the same one used for drawing the air). The insect was exposed to the volatiles for 16 min, and every 2 min the position of the olfactometer was rotated by 90°. The time spent and number of entries by *D. citri* in the different arms of the olfactometer was recorded using OLFA software (F. Nazzi, Udine, Italy). Ten replicates were done for each treatment. For each replicate, the olfactometer was changed, and a new insect was used. The olfactometer was set up in an aluminium cage (60 × 60 × 76 cm) provided with two fluorescent light tubes (70 W Luminux) positioned approximately 45 cm above the olfactometer. Two series of four-arm bioassays were carried out. In the first series of experiments, a volatile extract of each plant species was tested against n-hexane as control. The following bioassays were conducted: (i) volatile extract of *C. sinensis* versus n-hexane, (ii) volatile extract of *M. paniculata* versus n-hexane, (iii) volatile extract of *C. limettioides* versus n-hexane, (iv) volatile extract of *P. trifoliata* versus n-hexane, (v) volatile extract of *C. reshni* versus n-hexane, (vi) volatile extract of *P. guajava* versus n-hexane, (vii) volatile extract of *A. occidentale* versus n-hexane, and (viii) volatile extract of *M. indica* versus n-hexane. One microliter of volatile extract containing the plant volatile compounds or *n*-hexane was added to a filter paper strip and the solvent allowed to evaporate for 30 s before introducing the strips into individual arms. In the second series, responses of female *D. citri* to volatile extracts from *C. sinensis* and *M. paniculata* versus a synthetic blend of (*E*)-4,8-dimethylnona-1,3,7-triene (DMNT) and (*E*,*E*)-4,8,12-trimethyltrideca-1,3,7,11-tetraene (TMTT) were tested. The synthetic solution comprised 0.76 ng/ul of DMNT and 0.43 ng/ul of TMTT, using a similar level of these compounds identified in headspace VOCs obtained from *A. occidentale* plants (Table 1). The following treatments were evaluated: (i) volatile extract of *C. sinensis* (1 μl) vs. *n*-hexane (1 μl), (ii) volatile extract of *C. sinensis* (1 μl) + synthetic blend containing DMNT and TMTT (1 μl) vs. *n*-hexane (2 μl), and (iii) synthetic blend containing DMNT and TMTT (1 μl) vs. *n*-hexane (1 μl). The same set of bioassays was conducted by replacing the volatile extract of *C. sinensis* with the volatile extract of *M. paniculata*. The bioassays with extracts containing VOCs of *C. sinensis* and *M. paniculata* compared to *n*-hexane were conducted again, because the extracts used were not the same as those used in the first set of bioassays.

**Table 1:** Mean ± standard error (in μg/ 24 h) of volatile compounds of *Murraya paniculata*, *Citrus sinensis* and *Anacardium occidentale* calculated from 5 samples obtained by dynamic headspace collection during 24 h

Compounds	Retention index	*M. paniculata*	*C. sinensis*	*A. occidentale*
α-pinene	939	63.47 ± 29.08a	10.34 ± 1.59ab	3.63 ± 0.65b
camphene	953	7.14 ± 4.71a	10.68 ± 7.28a	1.22 ± 0.27a
6-methyl-5-hepten-2-one	985	29.26 ± 20.87a	11.15 ± 10.52a	1.60 ± 0.48a
myrcene	991	–	1.08 ± 0.49a	1.27 ± 0.29a
octanal	1003	12.12 ± 6.99ab	5.75 ± 3.17b	120.14 ± 39.7a
(*Z*)-3 hexenyl acetate	1011	37.03 ± 9.71a	19. 14 ± 7.53a	14.79 ± 7.71a
2-ethyl-1-hexanol^ab^	1029	54.24 ± 22.26a	77.95 ± 50.06a	32.25 ± 13.22a
limonene	1034	58.55 ± 16.21a	61.66 ± 29.99a	31.79 ± 11.53a
(*Z*)-ocimene	1038	–	7.02 ± 2.41a	8.59 ± 5.84a
(*E*)-ocimene	1049	6.12 ± 1.95a	663.53 ± 423.25b	15.03 ± 8.09a
linalool + undecane^c^	1100	72.52 ± 19.63a	129.54 ± 57.58a	78.87 ± 13.25a
nonanal	1105	126.33 ± 61.08a	88.74 ± 49.61a	80.63 ± 41.56a
DMNT^*^	1115	–	13.79 ± 6.21b	55.15 ± 19.59a
*(E)*-3-hexenyl butyrate^a^	1186	–	–	13.33 ± 4.48
methyl salicylate	1195	9.37 ± 3.45a	11.84 ± 5.03a	24.49 ± 13.82a
decanal	1207	627.91 ± 439.65a	216.91 ± 115.5a	373.37 ± 157.26a
benzothiazole^b^	1231	–	–	61.41 ± 46.28
indole	1294	–	27.21 ± 23.88	–
tridecane	1300	90.38 ± 39.03a	63.01 ± 14.05a	98.39 ± 36.36a
*cis*-jasmone	1394	–	14.64 ± 4.40	–
cyperene^a^	1410	–	–	48.34 ± 18.16
(*E*)-caryophyllene	1427	–	2.27 ± 0.77a	2.52 ± 0.72a
geranylacetone	1448	–	773.17 ± 558.73b	12.63 ± 3.71a
pentadecane	1500	–	85.83 ± 26.95a	109.81 ± 26.94a
(*E*,*E*)-α-farnesene	1504	–	–	49.55 ± 17.35
TMTT^*^	1574	–	3.83 ± 2.06b	31.37 ± 13.91a

^1^Means followed by the same letter within a line are not significantly different

^*^DMNT = (*E*)-4,8-dimethylnona-1,3,7-triene, TMTT = (*E*,*E*)-4,8,12-trimethyltrideca-1,3,7,11-tetraene

^a^Tentative identification

^b^Known contaminants of air entrainment samples

^c^Only detected in *M. paniculata*

### GC and Coupled GC-MS Analysis

Chemical analysis was performed for volatile extracts collected from *M. paniculata*, *C. sinensis,* and *A. occidentale*. Extracts were analysed on a Hewlett-Packard 7890A GC equipped with splitless injector and flame ionization detector (FID) using a non-polar DB-5MS bonded phase fused silica capillary column (30 m × 0.25 mm i.d., film thickness 0.25 μm). The oven temperature was maintained at 50 °C for 1 min, programmed at 5 °C min/min to 180 °C, and held for 0.1 min, then 10 °C min/min, to 250 °C. For quantitative analysis, the extracts were concentrated under a nitrogen flow to 50 μl, and 1 μl of tetracosane was added as internal standard (IS) for a final concentration of 0.02 mg/ml of the IS. One microliter of each sample was injected using a splitless mode with helium as carrier gas. Amounts released by the plant in each 24 h period were calculated in relation to the area of the internal standard. Data were collected with EZChrom Elite software and were handled using Excel (Microsoft Office 2007, Microsoft Corporation, USA). For qualitative analysis, selected extracts were analysed using a 5795MSD Agilent instrument equipped with a quadrupole analyser, a non-polar DB-5MS column (30 m × 0.25 mm ID, 0.25 μm film, J&W Scientific, Folsom, CA, USA) and a splitless injector, with helium as the carrier gas. Ionization was by electron impact (70 eV, source temperature 280 °C). Data were collected and analysed with ChemStation 2.03 Software (Agilent, USA). Tentative identifications were made by comparison of spectra with either library databases (NIST 2008) or with published spectra and were confirmed using authentic standards when available.

### Chemicals

Super Q (80/100 mesh) was purchased from Alltech (PA, USA). *n*-Hexane (95%, for pesticide residue analysis) was purchased from Fisher Scientific (Loughborough, Leicestershire, UK). (*Z*)-3-Hexenyl acetate (98%) were purchased from Alfa Aesar (Heysham, UK). Limonene (97%), *cis*-jasmone (92%) and linalool (96%) were purchased from TCI America (Portland, USA). α-Pinene (98%), camphene (90%), 6-methyl-5-hepten-2-one (98%), methyl salicylate (99%), ocimene (90%) (mix of (*E*) and (*Z*)-isomers), (*E*)-caryophyllene (80%), benzothiazole (96%), indole (98%), myrcene (97%), octanal (99%), undecane (99%), tridecane (99%), pentadecane (99%), nonanal (95%) and decanal (98%) were purchased from Sigma Aldrich (Steinheim, Germany). Geranylacetone (96%) was purchased from TCI (Tokyo, Japan). (*E*,*E*)-α-Farnesene was synthesized in three steps from isoprene and sulfur dioxide (Hassemer et al. 2016). (*E*)-4,8-Dimethyl-1,3,7-nonatriene (DMNT) and (*E*,*E*)-4,8,12-trimethyl-1,3,7,11-tridecatetraene (TMTT) were synthesized from geraniol and (*E*,*E*)-farnesol, respectively (Leopold 1990).

### Statistical Analysis

Collected olfactometer data, ie. the residence time (time spent in minutes) and the number of entries of *D. citri* in the treatment and control arms, were subjected to paired *t*-tests (*P* < 0.05), as described previously (Hegde et al. 2011; Sobhy et al. 2017). For this, the residence time in the control arms was obtained as a mean value. Only data for responding insects were included in the analysis. Non-responding insects, ie. those that stayed more than 2 min in the central area of the olfactometer and those that remained inactive (displaying neither walking nor antennal movement) were not considered. All statistical analyses were conducted using Genstat software (Genstat 2008). Collected analytical data, ie. the chemical profiles of volatile compounds obtained from *C. sinensis*, *M. paniculata,* and *A. occidentale* were analysed for each individual compound by Generalized Linear Model (GLM) and Deviance Analyses with gamma distribution and inverse as link function. When applying the Deviance Analysis method, all data from plants were included, and this gave an indication of the differences in chemical profiles. When the analyses showed significant effects of the species on the amount of the compounds, means were compared using contrast analyses. Plants that did not produce a compound were not included in the contrast analyses, ie. analytical data were compared in pairs. These analyses were performed using the statistical program R 2.14.0 (R Development Core Team 2009).

## Results

### Olfactometry - Host Plants

**Fig. 1:**
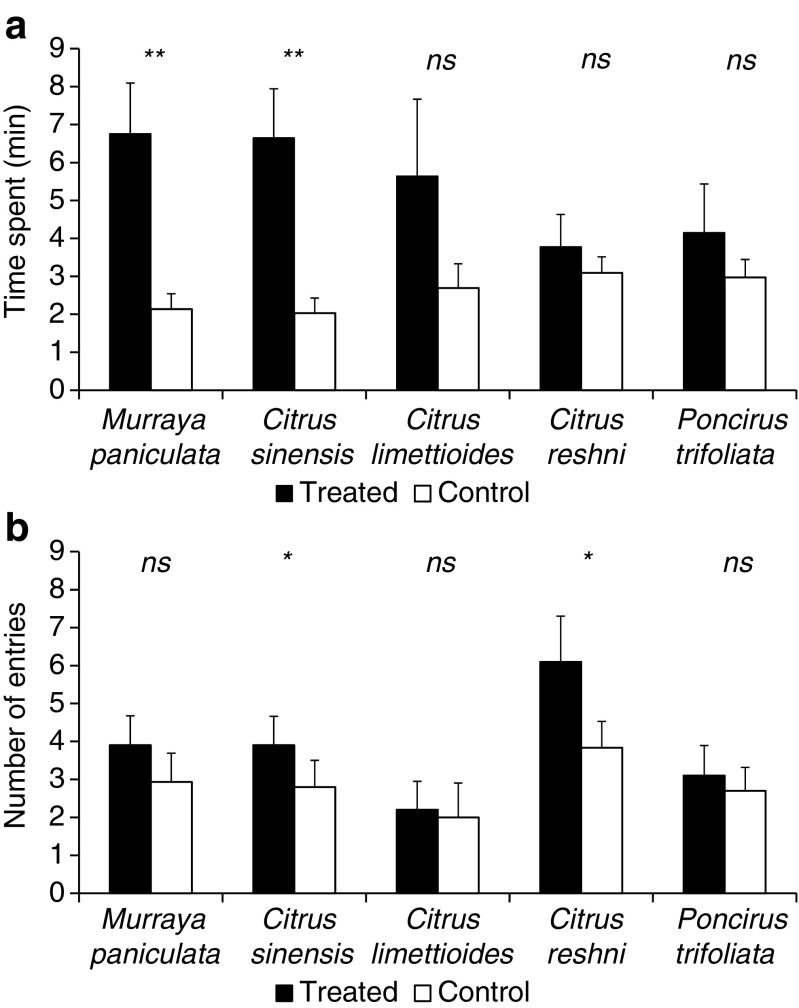
Time spent (**a**) and number of entries (**b**) (± s.e.) for *D. citri* females into treated (VOCs from host-plants) and control (*n*-hexane) arms

When comparing residence time between arms of the olfactometer with different treatments for each plant species, *D. citri* females spent more time in *M. paniculata* (*t* = 2.83; *df* = 9; *P* = 0.009) and *C. sinensis* (*t* = 2.97; *df* = 9; *P* = 0.007) treated arms compared to control arms (hexane solvent). There were no significant differences for the other host plants (*t* = 1.18; *df* = 9; *P* = 0.133, *t* = 0.60; *df* = 9; *P* = 0.281 and *t* = 0.74; *df* = 9; *P* = 0.239 for *C. limettioides*, *C. reshni,* and *P. trifoliata,* respectively) (Fig. 1a). There was a higher number of entries in the olfactometer arms treated with volatiles of *C. reshni* (*t* = 2.57; *df* = 9; *P* = 0.015) and *C. sinensis* (*t* = 1.84; *df* = 9; *P* = 0.049) in comparison with control olfactometer arms treated with *n*-hexane. Additionally, there was no significant effect for the other species (*t* = 1.35; *df* = 9; *P* = 0.105, *t* = 0.51; *df* = 9; *P* = 0.312, t = 0.98; *df* = 9; *P* = 0.175 for *M. paniculata*, *C. limettioides,* and *P. trifoliata*, respectively) (Fig. 1b).

### Olfactometry - Non-host Plants

**Fig. 2:**
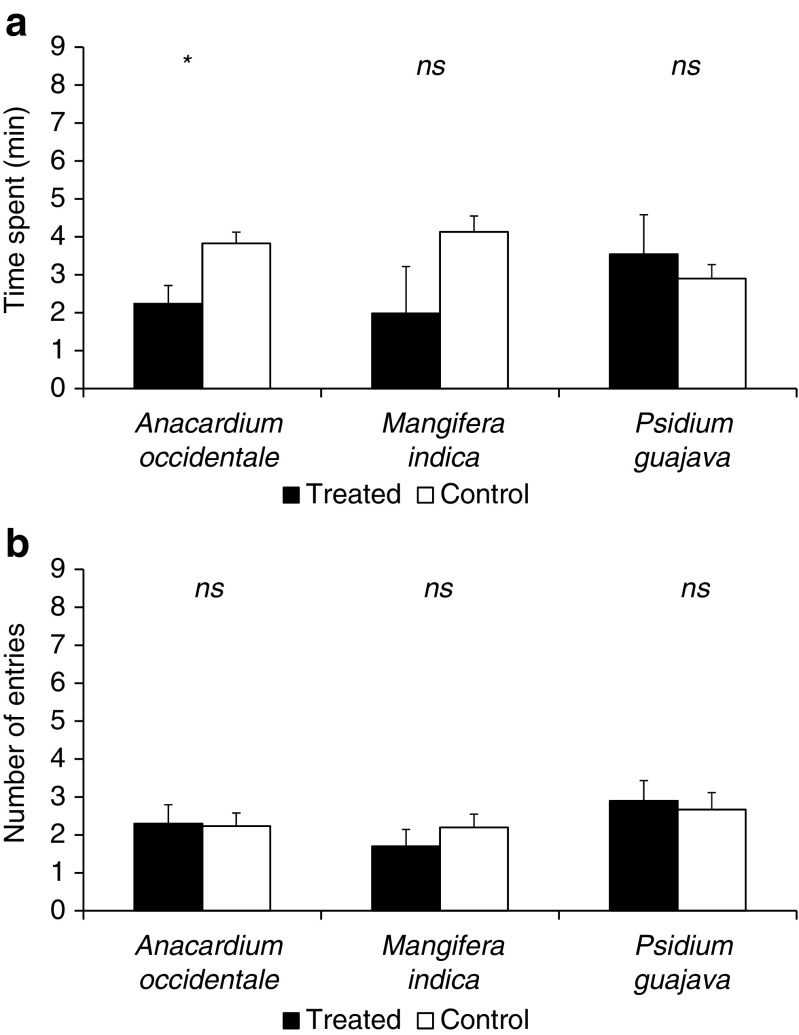
Time spent (**a**) and number of entries (**b**) (± s.e.) for *D. citri* females into treated (VOCs from non-host plants) and control (*n*-hexane) arms

When *D. citri* was exposed to a volatile extract from *A. occidentale*, they spent significantly more time in the control arm (*t* = −2.38; *df* = 9; *P* = 0.021) (Fig. 2a). However, there was no significant difference in the residence time when the volatile extracts from other non-host plants were tested against *n*-hexane (control) (*t* = −1.42; *df* = 9; *P* = 0.094 and *t* = 0.51; *df* = 9; *P* = 0.311 for *M. indica* and *P. guajava*, respectively) (Fig. 2a). Regarding the number of entries in the treated arms of the olfactometer for non-host plant volatiles versus control, *D. citri* did not show any significant response (*t* = 0.12; *df* = 9; *P* = 0.452 for *A. occidentale*, *t* = −1.04; *df* = 9; *P* = 0.162 for *M. indica* and *t* = 0.57; *df* = 9; *P* = 0.291 for *P. guajava* (Fig. 2b).

### Chemical Analysis

Chemical analysis of volatiles collected from two host species, *C. sinensis* and *M. paniculata* and comparison with the non-host *A. occidentale* revealed statistically significant qualitative and quantitative differences (Table 1). *A. occidentale* emitted a higher number of volatile compounds (Table 1). The deviance analysis showed differences in the amounts of the following compounds released by the plants: α-pinene (Anodev, *χ*^*2*^ = 7.92, *df* = 2, *P* < 0.02); camphene (Anodev, *χ*^*2*^ = 7.995, *df* = 2, *P* = 0.02), myrcene (Anodev, *χ*^*2*^ = 100.9, *df* = 2, *P* < 0.001), octanal (Anodev, *χ*^*2*^ = 24.654, *df* = 2, *P* < 0.001), (*Z*)-ocimene (Anodev, *χ*^*2*^ = 198.72, *df* = 2, *P* < 0.001), (*E*)-ocimene (Anodev, *χ*^*2*^ = 49.518, *df* = 2, *P* < 0.001), DMNT (Anodev, *χ*^*2*^ = 392,4, *df* = 2, *P* < 0.001), (*E*)-caryophyllene (Anodev, *χ*^*2*^ = 540.34, *df* = 2, *P* < 0.001), geranylacetone (Anodev, *χ*^*2*^ = 271.48, *df* = 2, *P* < 0.001), and TMTT (Anodev, *χ*^*2*^ = 264.32, *df* = 2, *P* < 0.001) (Table 1). The compounds *(E)-*3-hexenyl butyrate, benzothiazole, and the sesquiterpenes (*E*,*E*)-α-farnesene and cyperene (tentatively identified) were identified only in the volatile extract of *A. occidentale*. In addition, the contrast analysis showed that DMNT (*t* = 5.75, *df* = 12, *P* < 0.001) and TMTT (*t* = 2.42, *df* = 12, *P* = 0.03) were emitted in significantly higher amounts by *A. occidentale* compared to *C. sinensis* (Table 1), and octanal also was produced in higher amounts compared to *C. sinensis* (t = −2.13, *df* = 12, *P* = 0.05). The terpenoids DMNT and TMTT were not identified in volatile extracts from *M. paniculata* (Table 1). *cis*-Jasmone and indole were identified only in the volatile extracts of *C. sinensis*, whilst this species produced (*E*)*-*ocimene in significantly higher amounts compared to *A. occidentale* (*t* = 2.14, *df* = 12, *P* = 0.05) and *M. paniculata* (*t* = 2.15, *df* = 12, *P* = 0.05) and produced geranylacetone in higher amounts compared to *A. occidentale* (*t* = 2.48, *df* = 12, *P* = 0.03) (Table 1). *M. paniculata* produced α-pinene in higher amounts compared to *A. occidentale* (*t* = −2.13, *df* = 12, *P* = 0.05) (Table 1).

### Olfactometry - VOC Extracts and Synthetic Blends

**Fig. 3:**
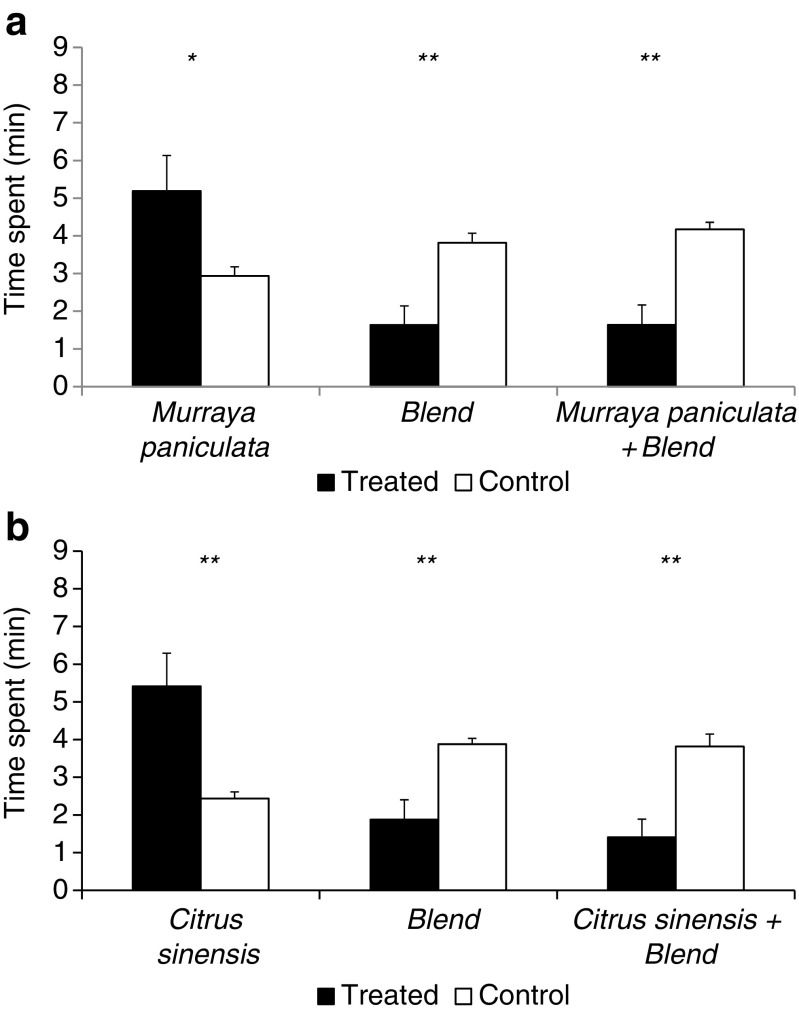
Time spent (± s.e.) for *D. citri* females into treated (*Citrus sinensis* air-entrainment extract) and control (*n*-hexane) arms, the synthetic blend versus control (*n*-hexane) and a combination of *C. sinensis* air-entrainment extract spiked with a synthetic blend (DMNT + TMTT) as treatments versus control (*n*-hexane) (**a**). Time spent (± s.e.) for *D. citri* females into treated (*M. paniculata* air-entrainment extract) and control (*n*-hexane) arms, the synthetic blend versus control (*n*-hexane) and a combination of *M. paniculata* air-entrainment extract spiked with a synthetic blend (DMNT + TMTT) as treatments versus control (*n*-hexane) (**b**)

**Fig. 4:**
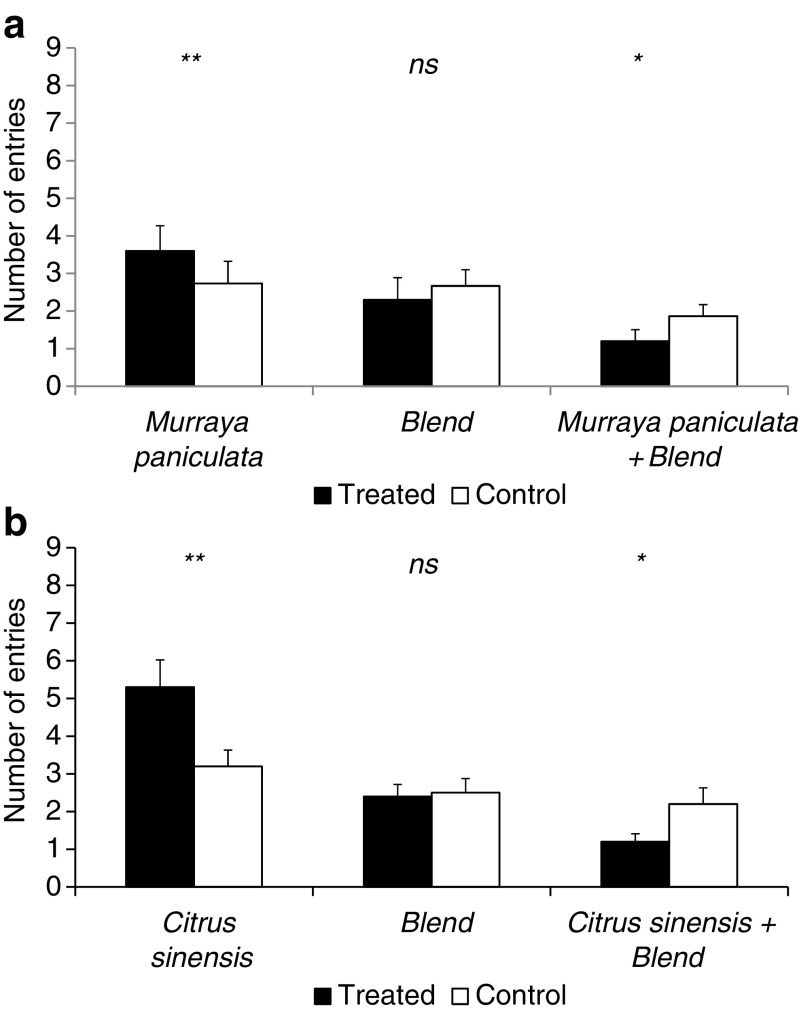
Number of entries (± s.e.) for *D. citri* females into treated (*Citrus sinensis* air-entrainment extract) and control (*n*-hexane) arms, the synthetic blend (DMNT + TMTT) versus control (*n*-hexane) and a combination of *C. sinensis* air-entrainment extract spiked with a synthetic blend as treatments versus control (*n*-hexane) (**a**). Number of entries (± s.e.) for *D. citri* females into treated (*M. paniculata* air-entrainment extract) and control (*n*-hexane) arms, the synthetic blend versus control (*n*-hexane) and a combination of *M. paniculata* air-entrainment extract spiked with a synthetic blend (DMNT + TMTT) as treatments versus control (*n*-hexane) (**b**)

In the second set of bioassays, *D. citri* females spent more time in the arm of the olfactometer containing volatiles of *M. paniculata* compared to the control (*n*-hexane) (*t* = 2.08; *df* = 9; *P* = 0.034) and in the arm containing volatiles from *C. sinensis* compared to *n*-hexane (*t* = 3,05; *df* = 9; *P* = 0.007) (Fig. 3a and b), confirming the result of the first set of bioassays. When the synthetic blend of DMNT and TMTT was evaluated versus *n*-hexane, *D. citri* residence time was higher in the olfactometer arms containing *n*-hexane (*t* = −3.51; *df* = 9; *P* = 0.003 and *t* = −3.72; *df* = 9; *P* = 0.002). When extracts from *M. paniculata* and *C. sinensis* were spiked with a synthetic blend of DMNT and TMTT, *D. citri* spent less time in the arm treated with the volatile extracts of both host plants + synthetic blend compared to *n*-hexane (*M. paniculata*, *t* = −3.97; *df* = 9; *P* = 0.002 and *C. sinensis*, *t* = −4.15; *df* = 9; *P* = 0.001), in the bioassays carried out for *M. paniculata* and *C. sinensis*, respectively) (Fig. 3a and b). *D. citri* females showed a higher number of entries in the olfactometer arm treated with *M. paniculata* (*t* = 4.99; *df* = 9; *P* < 0.001) and *C. sinensis* (*t* = 3.28; *df* = 9; *P* = 0.005) volatile extracts compared to *n*-hexane (Fig. 4a and b). There were no significant differences with regards to the number of entries into arms treated with the synthetic blend or *n*-hexane (*t* = −0.24; *df* = 9; *P* = 0.406 and *t* = −0.87; *df* = 9; *P* = 0.204). In the presence of the blend containing DMNT + TMTT combined with the attractive samples, insects showed a higher number of entries in the control arms (*n*-hexane) (*t* = −2,07; *df* = 9; *P* = 0.034 and *t* = −1,88; *df* = 9; *P* = 0.046 in the bioassays for *M. paniculata* and *C. sinensis*, respectively) (Fig. 4a and b).

## Discussion

In behavioural bioassays, female *D. citri* were significantly attracted to the volatiles collected by dynamic headspace collection from the host plants *M. paniculata*, *C. sinensis,* and *C. reshni* and were repelled by the volatiles emitted by the non-host plant *A. occidentale*. Thus, these compounds, present in greater amounts or solely found in *A. occidentale* volatiles, may be involved in the repellent behaviour i.e. (*E*)-3-hexenyl butyrate, benzothiazole, cyperene, (*E*,*E*)-α-farnesene and the two terpenoids DMNT and TMTT. The terpenoids have been reported as important plant defence volatiles involved in direct defence (i.e. as repellents) against a range of insect pests e.g. aphids, leafhoppers, stinkbugs (Hegde et al. 2011; Moraes et al. 2009; Oluwafemi et al. 2011; Sobhy et al. 2017), and in indirect defence via recruitment of natural enemy parasitic wasps (Bruce et al. 2008; Li et al. 2017; Moraes et al. 2009; Tamiru et al. 2011). Furthermore, Magalhães et al. (2012) suggested that cotton boll weevils, *Anthonomus grandis* recognize the physiological stage of host cotton plants via information on the amount of DMNT and TMTT released by cotton plants. Male and female *A. grandis* prefer plants that produce lower amounts of DMNT and TMTT, allowing discrimination between plants at the reproductive and vegetative stages (Magalhães et al. 2016). Although several putative repellent compounds were identified from *A. occidentale,* for the purpose of this study, we decided to investigate the activity of the terpenoids, given their reported activity for other insect pests and no reports of activity against psyllid bugs. The data presented here suggest that *D. citri*, as with other insect pests across different taxa, appear to avoid foraging on plants that produce significant amounts of these compounds, either in a constitutive or inducible fashion. Chemical analysis showed that *A. occidentale* produces higher amounts of DMNT and TMTT compared to *C. sinensis*, and these compounds were not identified in *M. paniculata* volatile extract. The volatiles emitted by *C. sinensis* and *M. paniculata* were attractive to *D. citri*, but when *D. citri* was exposed to volatiles from volatile extracts of either *C. sinensis* or *M. paniculata* combined with the synthetic mixture of DMNT and TMTT, *D. citri* preferred the control arm (*n*-hexane), and when these synthetic compounds were evaluated against *n*-hexane, the insects preferred the control arm containing the solvent *n*-hexane.

Although *C. reshni* volatiles were attractive to *D. citri,* based on the high number of entries, the insects did not spend significantly more time in the treated olfactometer arm. This behaviour could result in reduced oviposition, as reported by Tsagkarakis and Rogers (2010), who also observed a higher nymphal mortality in *C. reshni* ‘Cleopatra’ compared to *C. aurantium* under controlled conditions. *C. limettioides* volatiles were not attractive to *D. citri*. Tolerant to HLB under greenhouse conditions (Folimonova et al. 2009), this genotype did not favour adult populations under field conditions, but it is highly colonized by nymphs (Westbrook et al. 2011). In the present work, *P. trifoliata* was not found to be an attractive host, corroborating studies by Westbrook et al. (2011) under field conditions, who reported a low colonization rate on *P. trifoliata* accessions and Borgoni et al. (2014), who observed a low oviposition in this genotype under greenhouse conditions. There is evidence that selections obtained from this genotype may present differences on the degree of susceptibility to the insect but, besides that, this species might contribute to citrus breeding programs aimed at delivering resistance to *D. citri*.

The screening of volatile profiles of *D. citri* host plants can be useful for psyllid management and plant breeding for resistance to psyllids (Robbins et al. 2012) and may explain some differences in host plant suitability and disease susceptibility for the vector (Folimonova et al. 2009; Tsagkarakis and Rogers 2010; Westbrook et al. 2011). It is desirable, though, to correlate analytical data with biological studies to allow an understanding of insect-plant interactions and, consequently, the development of an integrated pest management system. On the other hand, the status of *M. paniculata* as a preferred host plant (Ikeda and Ashihara 2008), might contribute to the insect attractiveness, as stated in our results, and play an important role in the epidemiology and infection rates of HLB in citrus orchards. However, this effect can be altered in the presence of repellent volatiles, as was determined for *P. guajava* volatiles (Rouseff et al. 2008; da Silva et al. 2016; Zaka et al. 2010). In our study, a blend composed of DMNT and TMTT, combined with the VOCs from host plants, inhibited the foraging behaviour of *D. citri*, indicating that these chemicals could be involved in the lack of attraction of *D. citri* to non-host plants such as *A. occidentale* plants. However, the influence of other compounds identified only in non-host plants i.e. myrcene, benzothiazole, cyperene and (*E*,*E*)-α-farnesene should not be discounted. *C. sinensis* plants also produce the two terpenoids, but in very low amounts. Therefore, different genotypes of *C. sinensis* could be evaluated to verify if there are other varieties, including landraces that could produce these compounds in higher amounts and be less attractive to *D. citri*. Furthermore, molecular genetic approaches could be used to identify the functional genes involved in terpenoids biosynthesis in citrus plants, and new genotypes producing higher amounts of these secondary metabolites could be developed, thereby enhancing natural plant resistance against *D. citri*. Further work with genotypes and landraces is underway.
